# Do 6–9-year-old children in Denmark adhere to national dietary recommendations and are there sociodemographic disparities? The Generation Healthy Kids study

**DOI:** 10.1007/s00394-025-03863-y

**Published:** 2026-01-16

**Authors:** Frederik Holmegaard, Anna Gro Eilersen, Lotte Lauritzen, Christian Mølgaard, Ming Rong Liu, Ken D. Stark, Rikard Landberg, Rikke Fredenslund Krølner, Ulla Toft, Camilla Trab Damsgaard

**Affiliations:** 1 Department of Nutrition, Exercise and Sports, Faculty of Science, University of Copenhagen, Frederiksberg, Denmark; 2 Department of Kinesiology and Health Sciences, University of Waterloo, Waterloo, ON Canada; 3 Department of Life Sciences, Chalmers University of Technology, Gothenburg, Sweden; 4 National Institute of Public Health, University of Southern Denmark, Copenhagen, Denmark; 5 Department of Prevention, Health Promotion and Community Care, Steno Diabetes Center Copenhagen, Herlev, Denmark

**Keywords:** Children’s dietary intake, Dietary recommendations, Sociodemographic disparities, Public health

## Abstract

**Purpose:**

Diet in childhood is important for growth, brain development, and long-term health. Thus, assessing children’s adherence to dietary recommendations and identifying sociodemographic groups with low adherence is of great public health relevance. We investigated dietary intake, adherence to recommendations, and sociodemographic differences in a large cohort of Danish children.

**Methods:**

We analyzed baseline data from 1094 children aged 6–9 years from 23 schools across Denmark participating in the Generation Healthy Kids study. Diet was assessed by three-day dietary records and food frequency questionnaires for fish and supplements, focusing on key food groups, macronutrients, and iron. Fasting blood samples were collected from n = 347 and analyzed for nutritional biomarkers of fish, wholegrains, and iron for validation.

**Results:**

Overall adherence to dietary recommendations showed a mean ± SD score of 4.6 ± 1.0 out of 7.0. However, < 15% adhered to the recommendations for fruit + vegetables, fish, and meat, and < 33% to recommendations for saturated fat (SFA) and iron. Adherence decreased with age and shorter parental education, due to lower intakes of fruit + vegetables and dairy with age, and lower wholegrain and higher meat intake with shorter parental education. Also, rural children had lower adherence and consumed less fruit + vegetables and more added sugar and SFA than urban. Non-Danish descendants consumed less wholegrains and sugar than Danish, and weight status was not associated with adherence.

**Conclusions:**

Danish children had relatively good dietary adherence, but intakes of fruit + vegetables, fish, meat, SFA, and iron remain a concern. Attention should be given to children of older age, short parental education, and rural backgrounds.

**Supplementary Information:**

The online version contains supplementary material available at 10.1007/s00394-025-03863-y.

## Introduction

Diet and nutrient intake in childhood are important for optimal growth, cognitive and bone development, and long-term health [1–3]. In Western countries children from the age of 2 years are typically advised to follow the same food based dietary guidelines (FBDG) and nutrient recommendations as adults but adjusted to fit their lower total energy requirements [4–7]. Most dietary recommendations emphasize high intakes of fruit and vegetables, wholegrains, and fish while limiting red and processed meats, saturated fat (SFA), and added sugar. Such a dietary pattern has been shown to be associated with reduced overweight and improved cardiometabolic risk markers in children [8–11], which may influence later risk of lifestyle diseases. Thus, it is of great public health relevance to evaluate children’s adherence to dietary recommendations.

Previous dietary surveys have shown that children in Western countries have low adherence to dietary recommendations [4, 12]. A report from the European Food Safety Authority concluded that young children in the EU are at risk of insufficient dietary intakes of iron, vitamin D (vitD), and n-3 long-chain polyunsaturated fatty acids (n-3 LCPUFA) [13], a concern also evident among Danish children and adolescents [12]. However, the most recent national dietary survey among Danish children was conducted more than 10 years ago, and it did not include objective nutrient biomarkers, which are important to verify reported intakes and identify nutrient deficiencies.

Children’s dietary intake and adherence to recommendations may be influenced by sociodemographic factors. For instance, children of parents with lower education levels have been shown to consume less fruit and vegetables, fish, and dietary fiber than children of parents with higher education [14, 15], and adherence to the recommendations tends to decline as children approach adolescence [15, 16]. Children’s dietary intake may also be associated with other factors such as ethnic origin [17, 18] and rurality [19–21], although previous findings are inconsistent. As many sociodemographic factors are interrelated, comprehensive investigations that account for multiple factors simultaneously are needed to better understand social disparities in children’s food and nutrient intake. Finally, despite the common perception that children with overweight have unhealthy diets, studies on associations between children’s diets and weight status or adiposity remain inconclusive [15, 17, 22].

The present study used baseline data from 1094 6–9-year-old children from 23 schools in three of the five regions in Denmark, who participated in the Generation Healthy Kids (GHK) study. GHK was a cluster-randomized, multi-setting, multi-component intervention designed to promote healthy weight development in 1st and 2nd grade children by focusing on healthy diets, physical activity, sleep, and screen media habits [23].

The primary aim of the present study was to investigate the dietary intake of the children and their adherence to the Danish FBDG and Nordic Nutrition Recommendations (NNR2023). We focused on intake of fruit and vegetables, wholegrains, meat, milk and dairy, fish, SFA, added sugar, n-3 LCPUFA, vitD, and iron. In a subgroup of children, blood biomarkers were used to assess n-3 LCPUFA and iron status and to validate reported intakes of fish and wholegrains. Finally, we explored whether dietary intake and adherence differed by the children’s age, sex, parental education level, country of origin, rurality, and weight status.

## Methods

### Study design and participants

The present study is a cross-sectional investigation based on baseline data from the GHK study, collected during September-December 2023. The core components of the GHK intervention included a free-of-charge school lunch program, three weekly 40-min sessions of vigorous physical activity during school hours, and classroom exercises and parent workshops on sleep and screen media use, as previously described [23]. The study was approved by the Ethics Committee of Southern Denmark (S-20220094) and registered at clinicaltrials.gov (NCT 05940675).

Initial contact was established with 496 Danish schools from December 2022 to April 2023. Inclusion criteria for schools were: 1) location in The Capital Region, the Zealand Region, or the Region of Southern Denmark, 2) suitable kitchen facilities for school meal preparation. A total of 24 schools were recruited and randomized in clusters 1:1 to the intervention and control. One control school withdrew after randomization and 2006 children in 1st and 2nd grade were invited from the remaining 23 schools. There were no exclusion criteria for the children; if parents, teachers, or the clinically responsible physician (CM) judged that a child could not participate in certain parts of the intervention or measurements, they could still participate in the rest of the study. All children received oral information, and parents received both written and oral information about the study. Written informed consent for participation was obtained from the parents of 1350 children. Among these, 517 children (from the Capital and Zealand Regions only) also provided separate consent for blood sampling. The present study included the 1094 participating children who had ≥ 1 complete day of dietary recording and measurements of height and weight at baseline (Supplementary Fig. 1).

### Background information

The children’s age was collected from school records, and parents reported their education level and country of origin in an online questionnaire. Education level was reported in seven categories using the International Standard Classification of Education (ISCED97) adapted to a Danish context; 1) Primary education (1–6 years), 2) Lower secondary education (7–10 years), 3) Upper secondary education in high school (10–13 years), 4) Upper secondary vocational education (10–13 years), 5) Short tertiary education (14–16 years), 6) Bachelor’s degree or similar (14–17 years), and 7) Master’s degree or PhD (18–21 years). These categories were pooled into Short (≤ 13 y), Medium (14–17 y), and Long (≥ 18 y) education, and we used the highest level of education in the household for analyses. Children were classified as Danish descendants if one or both parents were born in Denmark, and as non-Danish descendants if both parents were born in other countries. Most non-Danish descendants originated from Eastern Europe (44%) and the Middle East (26%). When data was only available from one parent (n = 48), education level and descendance were based on this information. Children’s rurality was based on the geographical location of the schools, i.e. urban when located in cities or areas with ≥ 10,000 inhabitants or rural when located in towns or areas with < 10,000 inhabitants.

### Dietary records

Information about the children’s diet was collected sequentially at each school during the same period as the clinical measurements, but never on the same day. Parents were asked to record their child’s dietary intake prospectively for three consecutive days (two weekdays and one weekend day) using an online self-administered dietary assessment tool (myfood24®, Dietary Assessment Ltd., Leeds, UK).). 24-h dietary recalls in myfood24 have previously been validated against an interviewer-administered 24-h dietary recall in English adolescents [24] and against weighed dietary records and biomarkers in German adults [25]. Each day, parents received a link to the food diary with written guidelines in multiple languages and a video guide in Danish and English, and they had access to support by phone and e-mail. If all three days were not completed, reminder e-mails and text messages were sent to the parents 1–2 weeks later, on the same day(s) of the week.

Parents entered all food and drink items consumed by their child by searching a joint food database consisting of a Danish (FRIDA), a Swedish (Livsmedelsverket), an English (myfood24), and an American (USDA FoodData central) food composition database. Portion sizes were estimated using portion images, descriptions, household measures, or by weighing if the parents wished so.

### Estimation of dietary intake

From the dietary records, daily intakes of the food groups: fruit and vegetables, wholegrains, milk and dairy products, as well as meat and meat products were calculated. For mixed dishes, the contribution from each food group was estimated based on common recipes (e.g. for a stir fry with vegetables 50% was estimated to be vegetables). Wholegrain content was calculated using food specific wholegrain factors (e.g. 0.25 for wholegrain bread and buns, and 0.54 for wholegrain muesli and other breakfast cereals) based on the definitions of the Danish wholegrain labelling [26]. Daily intakes of SFA, dietary fiber, and iron were derived directly from myfood24. Intakes of added sugar were estimated as total sugar minus natural sugars (e.g. lactose in dairy) across the categories: sugar and jam, confectionary and chocolate, cakes and desserts, soft drinks, sauces and dips, refined grains, breakfast cereals, and dairy. For food items with missing database information on sugar content, added sugar were estimated from the Danish food composition database (FRIDA) [27] or product labels.

### Under-, acceptable, and over-reporters of dietary intake

Children were classified as either under-reporters, acceptable reporters, or over-reporters of energy based on the ratio of mean reported energy intake (EI) to estimated basal metabolic rate (BMR) using the cut-offs suggested by Black [28]; under-reporters: EI:BMR < 1.09, acceptable reporters: 1.09 < EI:BMR < 2.26, and over-reporters: EI:BMR > 2.26, assuming a physical activity level of 1.6 [7]. BMR was calculated from the Oxford equations [29] using the child’s height, weight, sex, and age.

### Food frequency questionnaire

To better assess habitual fish and n-3 LCPUFA intake, we used a self-administered, online food frequency questionnaire (FFQ) that asked specifically about the intake of fish. This fish FFQ was based on a semi-quantitative FFQ developed to assess overall food, energy, and nutrient intake in Denmark [30]. The original FFQ was validated in the Danish Cancer Cohort [31], and the derived fish specific FFQ has previously been validated among children in the FiSK trial [32].

Parents reported the overall frequency of their child’s fish intake during the last month as bread topping and as hot meals, separately, along with intake frequencies for 11 and 13 specific fish types within each meal type, respectively. Each question had seven response options ranging from “rarely or never” to “twice or more per day”, which were converted to average daily frequencies. To avoid overestimation, we multiplied the daily frequencies for each fish type with a correction factor, calculated as the recorded frequency of overall fish intake divided by the sum of frequencies for intake of all fish types. Average daily intakes of each fish type were then estimated by multiplying the adjusted frequencies with fish-specific portion sizes (30–50 g for bread toppings and 50–125 g for hot meals) based on validated Danish standard portion sizes [33]. Daily intakes of total, oily, and lean fish were calculated, and intakes of n-3 LCPUFA from fish were estimated using nutritional data from FRIDA.

The FFQ also included questions about frequency, type, and brand of n-3 LCPUFA and vitD supplements used in the past month and during winter (October–April), respectively. Daily intake of n-3 LCPUFA from supplements was estimated and added to the intake from fish to derive total daily intake. Children were categorized as vitD supplement users during winter when they consumed supplements with ≥ 10 µg of vitD on ≥ 5 days per week.

### Comparison with dietary recommendations

Children’s dietary intake was compared with the official dietary recommendations in Denmark at the time of data collection: the Danish FBDG 2021 and the NNR2023. The FBDG provide recommended intakes of food groups in a 10 MJ diet, i.e. ≥ 600 g/d fruit and vegetables, ≥ 75 g/d wholegrains, ≥ 350 g/week fish of which ≥ 200 g should be oily fish, ≥ 250 g/d milk and dairy, and ≤ 350 g/week meat and meat products. To compare directly with the FBDG and account for differences in EI, all food group intakes except fish and dairy were converted to g/10 MJ. For fish the recommended intake for 2-10-year-olds is 175-275 g/week, which we defined as 245 g/week for our cohort (6-9 years), and for dairy, Danish children are recommended to consume the same amount as adults despite a lower EI [6]. Nutrient intakes were converted to E% and g/MJ, as appropriate, for comparison with the NNR2023, e.g. < 10 E% added sugar, < 10 E% SFA, ≥ 2 g/MJ dietary fiber, and ≥ 9 mg/d iron [7].

To assess overall adherence to the dietary recommendations, a dietary adherence score was constructed based on seven key components: fruit and vegetables, wholegrains, fish, milk and dairy products, meat and meat products, added sugar, and SFA. For each component, a continuous score was calculated as the ratio of reported to recommended intake, capped at 1 for intakes meeting or exceeding the recommendations. For moderation components, i.e. meat, added sugar, and SFA, the scores were inverted, so lower intakes resulted in higher scores, and the score 0 was assigned to intakes at or above the 90th percentile of the distributions reported for 4–10-year-olds in the latest national dietary survey [12], similar to the approach used in the Healthy Eating Index [34]. The overall adherence score was calculated as the sum of scores for all components, ranging from 0 to 7, with higher scores indicating better adherence to the dietary recommendations.

### Clinical measurements and blood sampling

Clinical measurements and blood sampling were conducted in accordance with standard operating procedures on two separate school days by trained members of the research team. On the first day, children’s height and weight were measured. Height was determined as the mean of three measurements to the nearest 0.1 cm using a portable stadiometer (Seca 217, Seca, Germany), and weight was measured to the nearest 0.1 kg using a digital scale (InBody 270, InBody Co Ltd., California, USA). Children wore light clothing and were instructed to empty their bladder before being weighed. Sex- and age-adjusted z-scores for body mass index (BMI) were calculated using the WHO AnthroPlus software (v1.0.4, Department of Nutrition, WHO, Geneva, Switzerland) and the International Obesity Task Force (IOTF) LMS-curves [35]. Children were categorized as underweight, normal weight, and overweight as determined by age- and sex-specific cut-offs [35] for the IOTF BMI z-score.

On the second day, blood samples were drawn from an antecubital vein before 10:30 am after an overnight fast. In total, 347 children had a blood sample drawn and 325 of them (93.7%) confirmed fasting except for 1–2 glasses of water. Local anesthetic patches (EMLA, Astra Zeneca AB, Södertälje, Sweden) were provided beforehand and applied by the parents in the morning.

Whole blood for fatty acid analysis was collected with lithium heparin, and 500 µL was transferred to a cryotube with the antioxidant 2,6-di-tert-butyl-4-methylphenol (butylated hydroxytoluene; Sigma-Aldrich, St. Louis, MO, USA) and kept on dry ice. Additional blood collected in tubes with EDTA or clot activator was kept at room temperature and centrifuged at 2000 × g at 4 °C for 10 min within 6 h after blood sampling at the Department of Nutrition, Exercise, and Sports (NEXS), University of Copenhagen. All samples were stored at -80 °C until analysis.

### Analysis of blood samples

*Whole blood EPA* + *DHA:* Whole blood fatty acid composition was analyzed within 7 months by gas chromatography at the Department of Kinesiology and Health, University of Waterloo, ON, Canada, as previously described [36]. Briefly, lipids were extracted with chloroform and methanol containing an internal standard (22:3n-3 ethyl ester, Nu-Chek Prep Inc., Elysian, MN), and fatty acid methyl esters were generated using 14% boron trifluoride (BF_3_) in methanol and heating for 1 h at 95 °C on a heating block. The fatty acid methyl esters were then determined using a Scion 8300 gas chromatograph (Scion Instruments Canada, Edmonton, AB, Canada) with a DB-FFAP capillary column (15 m × 0.1 mm ID × 0.1 µm film thickness; J&W Scientific from Agilent Technologies, Mississauga, ON, Canada). In the initial analysis, some samples had very low fatty acid concentrations (< 0.5 µg/mg) with high SFA:LCPUFA ratios (> 6.0), indicating sample oxidation, as previously described [37]. Therefore, all samples were reanalyzed or reintegrated, and the results with the lowest SFA:LCPUFA ratio for each sample were included in the statistical analysis. Samples with SFA:LCPUFA ratios > 1 SD above or below the mean in both runs (n = 15, 4%) were excluded. The relative whole blood content of Eicosapentaenoic Acid (EPA) and Docosahexaenoic Acid (DHA) as a percentage of all fatty acids (FA%) was used as a measure of n-3 LCPUFA status. To allow comparison with omega-3-index cut-offs (< 4% [undesirable], 6%, and > 8% [optimal]) [38], whole blood EPA + DHA was converted to red blood cell equivalents, as suggested by Stark et al. [39].

*Plasma alkylresorcinols:* Plasma alkylresorcinol concentrations were analyzed at the Department of Life Sciences at Chalmers University of Technology, Sweden by liquid chromatography and tandem mass spectrometry on a QTRAP 6500+ (AB SCIEX, Marlborough, MA, USA), as previously described [40]. Total alkylresorcinol concentration – a marker of wholegrain wheat and rye intake – was used as a biomarker for validating the reported wholegrain intake. The intra- and inter-assay variability were 4.7–7.8% and 10.5–19.0% for each alkylresorcinol homologue, respectively.

*Iron status:* Whole blood hemoglobin was analyzed on a Sysmex KX 21N Analyzer (Sysmex Corporation, Hyogo, Japan) at NEXS within 6 h after blood sampling. Serum ferritin was analyzed on an IMMULITE 2000 Analyzer (Siemens Healthcare GmbH, Erlangen, Germany) at the Department of Clinical Biochemistry at Rigshospitalet, Copenhagen within one year after sampling.

### Statistical analysis

Data were analyzed using R (version 4.2.1). Crude odds ratios (OR) and a logistic regression model for age were used to assess sociodemographic differences between children included and not included in the present study. The sociodemographic characteristics of included children were compared to those of the Danish background population using chi-square tests.

Dietary intake, nutrient status, and adherence to dietary recommendations are presented as mean ± SD for normally distributed data and median (IQR) for skewed data. All mean and median intakes include children with zero intakes. Food and nutrient intakes were compared between boys and girls by Student’s unpaired t-test and Mann–Whitney U-test when data were normally distributed and skewed, respectively.

Children with acceptable dietary reporting and data on parental education and country of origin were included in the primary multivariable linear regression models to explore associations between sociodemographic factors (age, sex, weight status, parental education level, country of origin, and rurality) and intakes of key food groups and nutrients. The dietary components included in the models – fruit and vegetables, wholegrains, fish, milk and dairy products, meat and meat products, added sugar, and SFA – represent major targets for dietary improvement among children and are linked to both potential nutrient inadequacies and chronic disease prevention goals. All sociodemographic factors were included as fixed effects to assess the independent association of each predictor with dietary intakes. In secondary sensitivity analyses, under-reporters and over-reporters were included in all models first, and subsequently children with unanswered parental education and ethnic origin were included. Among the subgroup with blood samples, models of nutrient biomarkers were used as sensitivity analyses for associations with wholegrains and fish. Model assumptions were evaluated by visual inspection of Q-Q plots and scatter plots of residuals.

The FFQ-reported intakes of fish and n-3 LCPUFA and the registered intakes of wholegrains were validated against whole blood EPA + DHA and total plasma alkylresorcinols, respectively, using Spearman rank correlations. Furthermore, the dose–response relationships between estimated intakes of n-3 LCPUFA and wholegrains were fitted against their respective biomarkers using both linear and logarithmic regression. The resulting plots were compared by visual inspection and R^2^-values.

## Results

### Children’s characteristics and representativeness

Of the 1350 children originally included in GHK, 1142 children had at least one complete day of dietary recording at baseline. Ten were excluded from the analyses due to extreme dietary recordings (mean EI < 2000 or > 15,000 kJ/day), and 38 were excluded due to missing anthropometric measurements (Supplementary Fig. 1). The 256 non-included children were more likely to have parents with short education (OR, 95% CI 2.83, 1.75–4.61, *P* < 0.001), be non-Danish descendants (2.55, 1.58–4.04, *P* < 0.001), reside in urban areas (1.37, 1.03–1.83, *P* = 0.031) and have overweight (1.65, 1.09–2.45, *P* = 0.015), but did not differ with regard to sex and age (*P* > 0.65).

Boys and girls were equally represented among the included children (Table 1). Most children had normal weight, and the prevalence of underweight and overweight were comparable to the national numbers reported among Danish 6–7-year-olds (χ^2^ = 0.5, *P* = 0.795) [41]. Only 15.5% were from households with ≤ 13 years of education compared to 39.9% among all Danish families with children (χ^2^ = 12.3, *P* = 0.002) [42]. There was also a lower proportion of non-Danish descendants (7.8% vs. 16.2% among Danish 6–9-year-olds, χ^2^ = 43.5, *P* < 0.001) [43], whereas the proportion of children residing in urban areas was similar to the general population (60.1% vs. 58.3%, χ^2^ = 1.2, *P* = 0.265) [44].

**Table 1:** Characteristics of all children (n = 1094)

Demographics and anthropometrics	
Sex, % girls / % boys	48.8 / 51.2
Age, y	7.8 ± 0.6
Height, cm	130.3 ± 6.7
Weight, kg	26.7 (24.1–30.4)
BMI, kg/m^2^	15.7 (14.8–17.2)
BMI z-score	0.2 ± 1.1
Weight status	
Underweight	95 (8.7)
Normal weight	858 (78.4)
Overweight	141 (12.9)
Parental education level	
Short education (≤ 13 y)	170 (15.5)
Medium education (14–17 y)	468 (42.8)
Long education (≥ 18 y)	364 (33.3)
Unanswered	92 (8.4)
Origin	
Danish descendants	921 (84.2)
Non-Danish descendants	85 (7.8)
Unanswered	88 (8.0)
Rurality	
Urban	657 (60.1)
Rural	437 (39.9)
Dietary reporting	
Under-reporters	184 (16.8)
Acceptable reporters	886 (81.0)
Over-reporters	24 (2.2)

Data are presented as mean ± SD, median (IQR), or n (%) when data were normally distributed, skewed, or categorical, respectively

In total, 870 (80%), 128 (12%), and 96 (9%) children had three, two, and one complete dietary recording days, respectively, and more than 80% of the children (n = 886) were classified as acceptable reporters (1.09 < EI:BMR < 2.26). Over- and under-reporters were more likely to have parents with short education (OR, 95% CI 2.14, 1.37–3.32, *P* < 0.001), be non-Danish descendants (1.73, 1.03–2.82, *P* = 0.030), be boys (1.45, 1.08–1.96, *P* = 0.014), and have overweight (2.55, 1.73–3.74, *P* < 0.001).

### Dietary intake and nutrient status

Acceptable reporters consumed 7.0 ± 1.3 MJ/d (Table 2), which is within the estimated energy requirement range (6.3–7.8 MJ) for 6–9-year-old children with average physical activity levels (PAL = 1.6) according to NNR2023 [7]. Boys had higher EI than girls, but the relative macronutrient intakes were similar in boys and girls and aligned with the 45–60 E%, 25–40 E% and 10–20 E% recommended for carbohydrates, fat and protein [7], respectively. Boys had higher absolute intakes of wholegrains, dairy, meat, and iron, but did not differ from girls with regard to the other investigated food groups or n-3 LCPUFA intake (Table 2). In the subgroup with blood samples (n = 285), nutrient biomarkers did not differ by sex (Table 2). Iron deficiency (serum ferritin < 15 µg/L) was observed in 7.8% of the children, and 0.4% (n = 1) had anemia (hemoglobin < 6.5 mmol/L). n-3 LCPUFA status was generally low as 39.3% had an omega-3 index < 4% (undesirable), and only 0.4% had an index > 8% (optimal). Including over- and under-reporters did not change the results (Supplementary Table 1).

**Table 2:** Dietary intake (n = 886) and nutrient biomarkers (n = 264–283) of acceptable reporters

	Boys	Girls	Total
Energy and macronutrient intake			
Energy, MJ	7.3 ± 1.4	6.5 ± 1.2***	7.0 ± 1.3
Carbohydrates, E%	49.9 ± 5.5	50.4 ± 5.6	50.2 ± 5.6
Dietary fiber, g/MJ	2.7 ± 0.7	2.6 ± 0.7	2.7 ± 0.7
Added sugar, E%	8.0 (5.2–11.2)	8.8 (5.4–12.2)*	8.5 (5.4–11.7)
Fat, E%	32.9 ± 5.2	32.7 ± 5.0	32.8 ± 5.1
SFA, E%	11.2 ± 2.5	11.2 ± 2.5	11.2 ± 2.5
Monounsaturated fat, E%	11.2 ± 2.6	11.0 ± 2.4	11.1 ± 2.5
Polyunsaturated fat, E%	5.5 ± 1.4	5.4 ± 1.3	5.5 ± 1.4
Protein, E%	15.0 ± 2.9	14.8 ± 2.8	14.9 ± 2.8
Intake of food groups			
Fruit and vegetables, g/d	218 (130–327)	228 (143–326)	222 (137–326)
Wholegrains, g/d	53 (33–76)	39 (22–60)***	47 (28–70)
Fish^a^, g/d	12 (6–19)	12 (6–18)	12 (6–19)
Oily fish^a^, g/d	4 (0–8)	4 (1–7)	4 (1–7)
Milk and dairy products, g/d	217 (120–363)	188 (90–316)*	203 (104–343)
Meat and meat products, g/d	82 (52–124)	67 (44–101)***	75 (47–114)
Micronutrient intake			
n-3 LCPUFA^a^ (mg/d)	120 (42–242)	117 (54–231)	117 (44–238)
Iron (mg/d)	7.7 ± 2.0	6.7 ± 1.7***	7.2 ± 1.9
Food and nutrient biomarkers			
Total plasma alkylresorcinols^b^, nmol/L	275 (165–426)	234 (156–350)	248 (160–391)
Whole blood EPA + DHA^c^, FA%	3.2 ± 0.8	3.3 ± 0.8	3.3 ± 0.8
Whole blood hemoglobin^d^, mmol/L	8.1 ± 0.5	8.1 ± 0.6	8.1 ± 0.5
Serum ferritin^e^, µg/L	28 (21–38)	28 (21–40)	28 (21–39)

Data are presented as mean ± SD or median (IQR) when data were normally distributed or skewed, respectively

^a^Data from FFQ where n = 780,

^b^n = 264,

^c^n = 270,

^d^n = 277,

^e^n = 283

^*^P < 0.05, ***P < 0.001 for differences between boys and girls

### Validation of parent-reported wholegrain, fish, and n-3 LCPUFA intakes

In the subgroup with blood samples, there was a dose–response relationship between wholegrain intake and plasma total alkylresorcinols (Supplementary Fig. 2), and parent-reported wholegrain intake was moderately correlated with total alkylresorcinols (r = 0.33, *P* < 0.001).

Absolute intakes of fish, oily fish and total n-3 LCPUFA from fish and supplements were moderately correlated with whole blood EPA + DHA (r = 0.30–0.32, all *P* < 0.001), whereas the correlation for lean fish, which contains little n-3 LCPUFA, was weaker (r = 0.21, *P* < 0.05). The dose–response relationship between total n-3 LCPUFA intake and whole blood EPA + DHA was best fitted by a logarithmic function (Supplementary Fig. 3).

### Adherence to dietary recommendations

The average adherence score was 4.6 ± 1.0 out of 7.0, indicating that children’s overall adherence to the dietary recommendations was fairly good. However, as shown in Table 3, less than 15% of the children adhered to the dietary recommendations for fruit and vegetables and fish. The low intake of fish, especially oily fish, was in line with the low whole blood n-3 LCPUFA observed (Table 2). The adherence with recommendations for intakes of meat and SFA was also low, as less than one third of the children consumed less than 50 g/10 MJ and 10 E%, respectively. Almost half of the children met the recommendations for wholegrains and dairy, and more than 60% adhered to the recommendations for added sugar, dietary fiber, and vitD supplementation during winter (Table 3). Including children with over- and underreporting in the analyses did not change the results (Supplementary Table 2).

**Table 3:** Children’s adherence to dietary recommendations (n = 886)

Food group or nutrient	Danish FBDG or NNR2023	Daily intake	Adherence, n (%)
Fruit and vegetables, g/10 MJ	≥ 600 g/10 MJ	322 (196–472)	104 (11.7)
Wholegrains, g/10 MJ	≥ 75 g/10 MJ	68 (41–99)	394 (44.5)
Fish^a^, g/d	≥ 35 g/d (Min. 245 g/w)	12 (6–19)	56 (7.2)
Oily fish^a^, g/d	≥ 20 g/d (Min. 140 g/w)	3.7 (0.7–7.2)	46 (5.9)
Milk and dairy products, g/d	≥ 250 g/d	203 (104–343)	356 (40.2)
Meat and meat products, g/10 MJ	≤ 50 g/10 MJ (Max. 350 g/w)	110 (72–164)	125 (14.1)
SFA, E%	< 10 E%	11.1 (9.4–12.8)	288 (32.5)
Dietary fiber, g/MJ	≥ 2 g/MJ	2.7 ± 0.7	731 (82.5)
Added sugar, E%	< 10 E%	8.5 (5.4–11.7)	568 (64.1)
Iron, mg/d	≥ 8 mg/d	7.2 ± 1.9	272 (30.7)
VitD supplement during winter^b^	Supplement during winter (Oct-Apr)	487 yes, 307 no	487 (61.3)

Daily intakes are presented as mean ± SD, median (IQR), or n when data were normally distributed, skewed, or binomial, respectively

^a^Data was derived from the food frequency questionnaire, n = 780

^b^Data was derived from the supplements’ questionnaire, n = 794

### Sociodemographic determinants of dietary intake

In the primary multivariable linear regression models with acceptable reporters, overall adherence to dietary recommendations decreased with age (Table 4). This was mainly due to lower intakes of fruit and vegetables and dairy as well as tendencies to lower fish and higher sugar intake with age. The associations for fruit and vegetables, dairy, and fish were supported in the sensitivity analyses with inclusion of under- and over-reporters and those with missing sociodemographic data, but the significance of the association for sugar decreased somewhat (Supplementary Table 3 and 4). However, the sensitivity analysis among the subgroup with blood samples did not support a decline in fish intake with age, as whole blood EPA + DHA tended to increase with age (Supplementary Table 5).

**Table 4:** Associations between sociodemographic characteristics and dietary intake among children with acceptable dietary reporting and complete data on parental education and country of origin (n = 824)

	Fruit and vegetables, g/10 MJ		Wholegrains, g/10 MJ		Fish^a^, g/d		Milk and dairy, g/10 MJ		Meat and meat products, g/10 MJ		Added sugar, E%		SFA, E%		Adherence score	
	β (95% CI)	P	β (95% CI)	P	β (95% CI)	P	β (95% CI)	P	β (95% CI)	P	β (95% CI)	P	β (95% CI)	P	β (95% CI)	P
Sex																
Girls	Ref	< 0.001	Ref	< 0.001	Ref	0.727	Ref	0.943	Ref	0.091	Ref	0.008	Ref	0.409	Ref	0.230
Boys	-59.2 (-86.3, -32.1)		13.6 (7.7, 19.5)		-0.4 (-2.6, 1.8)		-1.3 (-36.4, 33.8)		8.2 (-1.3, 17.6)		-0.9 (-1.6, -0.2)		-0.1 (-0.5, 0.2)		0.1 (-0.1, 0.2)	
Age, y	-27.6 (-50.5, -4.7)	0.018	-4.1 (-9.1, 0.9)	0.110	-1.7 (-3.6, 0.1)	0.070	-67.7 (-97.4, -38.1)	< 0.001	2.8 (-5.2, 10.8)	0.492	0.5 (-0.1, 1.1)	0.075	-0.1 (-0.4, 0.2)	0.648	-0.2 (-0.3, -0.1)	0.002
Weight status																
Underweight	9.5 (-45.6, 64.5)	0.494	8.9 (-3.2, 20.9)	0.198	3.4 (-1.2, 8.0)	0.234	36.1 (-35.3, 107.6)	0.399	-15.6 (-34.8, 3.7)	0.168	0.0 (-1.4, 1.3)	0.811	0.3 (-0.5, 1.0)	0.557	0.1 (-0.2, 0.3)	0.585
Normal weight	Ref		Ref		Ref		Ref		Ref		Ref		Ref		Ref	
Overweight	-25.1 (-79.5, 29.3)		-1.9 (-13.8, 10.0)		0.0 (-4.4, 4.4)		-17.0 (-87.6, 53.7)		-2.3 (-21.3, 16.7)		-0.4 (-1.7, 1.0)		-0.2 (-0.9, 0.5)		-0.1 (-0.4, 0.2)	
Parental education																
Long	Ref	0.148	Ref	0.003	Ref	0.124	Ref	0.820	Ref	0.003	Ref	0.652	Ref	0.923	Ref	< 0.001
Medium	-23.2 (-59.3, 12.9)		-8.2 (-16.1, -0.4)		-2.1 (-5.0, -0.8)		-3.1 (-49.9, 43.8)		15.2 (2.6, 27.8)		0.0 (-0.8, 0.9)		-0.1 (-0.5, 0.4)		-0.2 (-0.4, -0.1)	
Short	-38.7 (-89.5, 12.0)		-15.2 (-26.3, -4.1)		-3.2 (-7.3, 1.0)		13.6 (-52.2, 79.5)		23.0 (5.3, 40.8)		0.5 (-0.8, 1.7)		0.0 (-0.7, 0.6)		-0.4 (-0.6, -0.1)	
Origin																
Danish	Ref	0.498	Ref	< 0.001	Ref	0.827	Ref	0.233	Ref	0.666	Ref	< 0.001	Ref	0.051	Ref	0.185
Non-Danish	17.5 (-33.2, 68.2)		-28.6 (-39.6, -17.5)		0.5 (-3.7, 4.6)		40.0 (-25.8, 105.8)		3.9 (-13.8, 21.6)		-2.5 (-3.7, -1.2)		0.6 (0.0, 1.3)		-0.2 (-0.4, 0.1)	
Residential area																
Urban	Ref	< 0.001	Ref	0.017	Ref	0.255	Ref	0.444	Ref	0.362	Ref	0.002	Ref	0.026	Ref	0.028
Rural	-52.9 (-81.7, -24.1)		7.7 (1.4, 13.9)		-1.4 (-3.7, 1.0)		14.6 (-51.9, 22.8)		4.7 (-5.4, 14.7)		1.1 (0.4,1.8)		0.4 (0.0, 0.8)		-0.2 (-0.3, 0.0)	

Regression coefficients (β), 95% confidence intervals (CI), and *P*-values were derived from multivariable linear regression models examining the independent associations of sex, age, weight status, parental education level, country of origin, and residential area with intake of selected food groups and nutrients, as well as an overall dietary adherence score.

^a^Fish intake data were derived from the food frequency questionnaire, n = 776

Longer parental education was associated with better overall adherence to the dietary recommendations, mainly due to higher intakes of wholegrains and lower intakes of meat. The association with wholegrain intake was supported by a positive association between parental education and plasma total alkylresorcinols (Supplementary Table 5). The sensitivity analyses including more children also supported the association with wholegrains (Supplementary Table 3 and 4), but they did not provide clear support for the association with meat.

Children residing in urban areas had higher adherence to the dietary recommendations than rural children. All analyses showed that this was due to a higher intake of fruit and vegetables and lower intakes of added sugar and SFA (Table 4; Supplementary Table 4 and 5). However, urban children consumed less wholegrains than rural children.

The overall adherence score did not differ by sex, weight status, or origin in the primary or any of the sensitivity analyses (Table 4; Supplementary Table 4 and 5). All of the analyses did, however, show that non-Danish descendants consumed less wholegrains, but also less added sugar than Danish descendants. Consistent associations were also seen for sex, with higher consumption of wholegrains and less added sugar, but a concomitantly reduced intake of fruit and vegetables among boys relative to girls. Weight status was not associated with intake of any of the food groups or nutrients in the primary analysis, but in the sensitivity analyses meat intake was higher, whereas added sugar and SFA intake tended to be lower with increasing weight status.

## Discussion

In a large group of Danish 6–9-year-olds, we showed that most children consumed less fruit and vegetables and fish as well as more meat than recommended, whereas almost half of the children reached the recommended level for wholegrain intake and more than 60% adhered to the recommendation for added sugar. Overall adherence to the dietary recommendations improved with higher parental education level and decreased with age. Children living in rural areas had lower adherence to the recommendations for fruit and vegetables, added sugar, and SFA, whereas the wholegrain intake was lower among urban children and Danish descendants.

The low intake of fruit and vegetables aligns with previous findings among Western children [12, 17, 45]. This is a concern as fruit and vegetables are key sources of micronutrients and dietary fiber and have been linked to lower risk of overweight [46] and improved cardiometabolic risk profile in children [10]. In contrast to the relatively high fish intake and n-3 LCPUFA status among children in Norway [47, 48], fish intake in the present study was very low as previously seen in Denmark [12, 32, 45], the US [4], and the UK [17]. This finding was supported by the low whole blood EPA + DHA which also aligned with previous reports in Danish children [32, 49]. Increasing the n-3 LCPUFA status of Danish children would be desirable, as this has been linked to better cognitive function [50] and a more favorable cardiometabolic risk profile [49], and both of these outcomes were found to be improved by an oily fish intake of ≈300 g/wk in our previous randomized trial in Danish children [8, 51]. The persistent low intakes of fruit and vegetables and fish may reflect barriers such as cost, accessibility, preferences, time constraints, and cooking skills [32, 52]. In contrast, intake of meat was high, as also seen in the latest national dietary survey [12], suggesting a continued predominance of animal-based foods in the Danish diet despite increased trends towards plant-based diets.

In general, wholegrain intake was high with a median intake approximately three times greater than among children in the UK and the US [4, 53]. Even though Denmark has one of the highest wholegrain recommendations globally [54], nearly half of the children met the recommendation, consistent with the latest national dietary survey [12]. This is likely beneficial for long-term health, as a high wholegrain intake has been shown to improve the plasma lipid profile in children [9] and to be linked to reduced risk of cardiovascular disease and mortality in adults [55]. Wholegrain intakes have increased substantially in Denmark since the early 2000’s [26], likely due to initiatives such as the Danish Wholegrain Partnership, which has improved the availability, labeling, and public awareness of wholegrain products. However, wholegrain intake among non-Danish descendants remained particularly low in the present study, with only 21.5% meeting the recommendation. One explanation may be that wholegrain products, such as sour dough rye bread and rolled oats, are distinctive characteristics of the Danish food culture, which may take time to adopt among immigrant populations. The Danish recommendation for wholegrains was raised to 90 g/10 MJ in June 2024 [56], and when compared to this update, 31.3% of the children still adhered.

Surprisingly, 64% of the children adhered to the recommendation of limiting added sugar to < 10 E%. This is a marked improvement compared to the 49% and 34% of 4–14-year-olds reported in the Danish national surveys from 2011–2013 and 2003–2008, respectively [12, 57]. Despite efforts to expand the myfood24 database with added sugar values to minimize underestimation, some underreporting either due to incomplete databases or parental reporting cannot be ruled out [58]. Nevertheless, similar downward trends in sugar intake have been observed in the US and Germany since the early 2000s, especially between 2010–2016 [59, 60]. However, unlike in the present study, children’s mean and median intakes of added sugar still exceeded the recommendation in these populations [59, 60].

The reported iron intake was somewhat low considering the children’s high meat consumption, and when compared to the recommended intake (8.0 mg/d for 7–8-year-olds [7]) and the latest national dietary survey (6.7 ± 1.7 mg/d vs. 8.9 ± 2.1 mg/d and 7.7 ± 2.0 mg/d vs. 9.6 ± 2.4 mg/d in 6–9-year-old girls and boys, respectively [12]). However, both hemoglobin and ferritin levels were within the recommended ranges and only 8% of the children had iron deficiency, suggesting that the intake was adequate for most children. The intake of iron might be underestimated, but the findings may also indicate that the high bioavailability of iron from meat, or increased iron absorption seen at low intakes [61], compensated for the low total intake.

Consistent with previous studies [14–16], adherence to dietary recommendations improved with higher parental education level and decreased with age. Similar to the present study, parental education level was also associated with wholegrain intake in the Danish national survey 20 years ago, although only among girls [14], and the observed tendency for reduced meat intake with longer parental education aligns with findings from the EU and the UK, particularly for processed meats [62, 63]. These associations may reflect greater health- and environmental awareness among highly educated parents. The decline in adherence to dietary recommendations with increasing age was evident despite a narrow age range in the present study [15, 16]. Early school years may be a period of rapid dietary changes, as children have to adapt to new environments and engage in new social relationships, which may prompt them to align their food preferences with perceived peer norms to ensure social acceptance [64].

The lower overall adherence to dietary recommendations, lower fruit and vegetable intake, and higher sugar intake among rural compared to urban children are consistent with studies from Europe [19] and Canada [20], whereas the higher SFA intake has not previously been reported. Rural children also had a higher wholegrain intake, which may reflect a stronger adherence to the Danish tradition of having wholegrain rye bread for lunch in rural areas, as also seen in Finland [65]. In contrast, an Australian study found that children residing in remote areas had overall healthier eating habits and consumed more fruit, and less takeaway foods and unhealthy snacks [21]. Besides differences in the definitions of urban and rural areas and the remoteness of the rural areas in the studies, the discrepancies could relate to country-specific differences in rural versus urban food environments.

A major strength of the present study was the large sample size of 1094 children which exceeded that of the national Danish dietary surveys [12]. We included participants from a wide geographic area across Denmark, who were representative of the general population in terms of sex, weight status, and residential area. Non-Danish descendants were better represented than in the national surveys [12], but they were still under-represented as were children from shorter educational backgrounds. This may have led to an overestimation of the adherence to the dietary recommendations and limited our ability to detect differences between groups, particularly by origin. The detailed dietary records of the whole diet combined with the fish-specific FFQ allowed for a comprehensive assessment of habitual dietary intake, which was validated against objective biomarkers. A three-day recording period was selected to ensure feasibility, particularly among less advantaged families, but it was not suited for evaluating micronutrient intake [66]. The online recording tool helped structure the recordings, had built-in reminders (e.g. for snacks and toppings), and allowed parents to edit entries during the day, thereby reducing reliance on memory. To support non-Danish families, we provided text instructions in Arabic and English and video instructions in English, and the software interface was available in 10 languages. However, the food item list remained in Danish which may have challenged families with other language backgrounds.

In conclusion, the overall adherence to the Danish FBDG and NNR2023 was relatively high in the examined large group of Danish 6–9-year-old children, but challenges remain particularly in terms of intakes of fruit and vegetables, fish, meat, SFA, and iron. Sociodemographic differences in dietary intake were especially related to children’s age, parental education level, and residential area, but not to weight status. These findings highlight the need for early interventions to improve children’s dietary quality while also considering strategies for specific sociodemographic groups where improvements are needed the most.

## Supplementary Information

Below is the link to the electronic supplementary material.

Supplementary file1 (DOCX 83 kb)

## Data Availability

The datasets analyzed in the current study are not publicly available due to ethical restrictions, but they are available from the corresponding author upon reasonable request.

## Abbreviations

BMR: Basal metabolic rate
DHA: Docosahexaenoic acid
EI: Energy intake
EPA: Eicosapentaenoic acid
FBDG: Food based dietary guidelines
FFQ: Food frequency questionnaire
FRIDA: The Danish food composition database
GHK: Generation Healthy Kids
NNR2023: Nordic Nutrition Recommendations
n-3 LCPUFA: n-3 long chain polyunsaturated fatty acids
SFA: Saturated fat
vitD: Vitamin D
