# Supplementary material for: Do 6–9-year-old children in Denmark adhere to national dietary recommendations and are there sociodemographic disparities? The Generation Healthy Kids study
Source: Eur J Nutr. 2026 Jan 16;65(1):24. doi: 10.1007/s00394-025-03863-y (PMC12811282; doi:10.1007/s00394-025-03863-y)
Supplement: Supplementary file 1 — Supplementary file1 (DOCX 83 kb) [file 394_2025_3863_MOESM1_ESM.docx]

Supplementary files

Do 6-9-Year-Old Children in Denmark Adhere to National Dietary Recommendations and Are There Sociodemographic Disparities? The Generation Healthy Kids Study

European Journal of Nutrition

**Authors**

Frederik Holmegaard^1^†, Anna Gro Eilersen^1^†, Lotte Lauritzen^1^, Christian Mølgaard^1^, Ming Rong Liu^2^, Ken D. Stark^2^, Rikard Landberg^3^, Rikke Fredenslund Krølner^4^, Ulla Toft^5^, Camilla Trab Damsgaard^1^

†These authors contributed equally to this paper and share first authorship.

**Affiliations**

^1^Department of Nutrition, Exercise and Sports, Faculty of Science, University of Copenhagen, Frederiksberg, Denmark

^2^Department of Kinesiology and Health Sciences, University of Waterloo, Waterloo, ON, Canada

^3^Department of Life Sciences, Chalmers University of Technology, Gothenburg, Sweden

^4^National Institute of Public Health, University of Southern Denmark, Copenhagen, Denmark

^5^Department of Prevention, Health Promotion and Community Care, Steno Diabetes Center Copenhagen, Herlev, Denmark

**Corresponding author**

Frederik Holmegaard, E-mail: [fhj@nexs.ku.dk](mailto:fhj@nexs.ku.dk)

| **Supplementary table 1.** Dietary intake of all participants (n=1094) and nutrient biomarkers (n=317-342) | | | | | | |
| --- | --- | --- | --- | --- | --- | --- |
|  | Boys |  | Girls |  | Total |  |
| **Energy and macronutrient intake** |  |  |  |  |  |  |
| Energy, MJ/d | 7.0 ± 1.7 |  | 6.1 ± 1.6*** |  | 6.6 ± 1.7 |  |
| Carbohydrates, E% | 50.1 ± 5.6 |  | 50.6 ± 6.1 |  | 50.3 ± 5.9 |  |
| Dietary fiber, g/MJ | 2.7 ± 0.7 |  | 2.7 ± 0.7 |  | 2.7 ± 0.7 |  |
| Added sugar, E% | 7.9 (5.0-11.2) |  | 8.7 (5.3-12.0) |  | 8.2 (5.2-11.5) |  |
| Fat, E% | 32.5 ± 5.4 |  | 32.2 ± 5.3 |  | 32.4 ± 5.4 |  |
| SFA, E% | 11.1 ± 2.7 |  | 11.1 ± 2.7 |  | 11.1 ± 2.7 |  |
| Monounsaturated fat, E% | 11.0 ± 2.7 |  | 10.7 ± 2.5 |  | 10.9 ± 2.6 |  |
| Polyunsaturated fat, E% | 5.5 ± 1.4 |  | 5.4 ± 1.4 |  | 5.4 ± 1.4 |  |
| Protein, E% | 15.2 ± 3.0 |  | 15.0 ± 3.1 |  | 15.1 ± 3.1 |  |
|  |  |  |  |  |  |  |
| **Intake of food groups** |  |  |  |  |  |  |
| Fruit and vegetables, g/d | 204 (123-320) |  | 218 (135-306) |  | 211 (129-313) |  |
| Wholegrains, g/d | 52 (31-75) |  | 38 (21-60)*** |  | 44 (26-68) |  |
| Fish^a^, g/d | 12 (6-19) |  | 12 (6-19) |  | 12 (6-19) |  |
| Oily fish^a^, g/d | 4 (0-8) |  | 4 (1-7) |  | 4 (1-8) |  |
| Milk and dairy products, g/d | 202 (106-346) |  | 176 (83-299)** |  | 197 (90-318) |  |
| Meat and meat products, g/d | 78 (47-119) |  | 63 (40-97)*** |  | 71 (43-109) |  |
|  |  |  |  |  |  |  |
| **Micronutrient intake** |  |  |  |  |  |  |
| n-3 LCPUFA (mg/d) | 107 (36-219) |  | 108 (39-206) |  | 108 (36-215) |  |
| Iron (mg/d) | 7.4 ± 2.2 |  | 6.4 ± 2.0*** |  | 6.9 ± 2.2 |  |
|  |  |  |  |  |  |  |
| **Food and nutrient biomarkers** |  |  |  |  |  |  |
| Plasma alkylresorcinols^b^, nmol/L | 270 (160-444) |  | 234 (163-369) |  | 250 (163-409) |  |
| Whole blood EPA+DHA^c^, FA% | 3.2 ± 0.8 |  | 3.3 ± 0.8 |  | 3.3 ± 0.8 |  |
| Serum ferritin^d^, µg/L | 28 (21-39) |  | 28 (21-41) |  | 28 (21-39) |  |
| Hemoglobin^e^, mmol/L | 8.1 ± 0.5 |  | 8.1 ± 0.5 |  | 8.1 ± 0.5 |  |
| Data are presented as mean±SD and median (IQR), when data were normally distributed and skewed, respectively.  **P<0.01, ***P<0.001 for differences between boys and girls  ^a^Data from FFQ where n=960,  ^b^n=317,  ^c^n=326,  ^d^n=342,  ^e^n=334 | | | | | | |

| **Supplementary table 2.** Children’s adherence to dietary recommendations (n=1094) | | | | | | | |
| --- | --- | --- | --- | --- | --- | --- | --- |
| Food group or nutrient |  | Danish FBDG or NNR2023 |  | Daily intake |  | Adherence, n (%) |  |
| Fruit and vegetables, g/10 MJ |  | ≥600 g/10 MJ |  | 325 (196-484) |  | 145 (13.3) |  |
| Wholegrains, g/10 MJ |  | ≥75 g/10 MJ |  | 70 (41-102) |  | 501 (45.8) |  |
| Fish^a^, g/d |  | ≥35 g/d (Min. 231 g/w)^c^ |  | 12 (6-19) |  | 72 (7.6) |  |
| Oily fish^a^, g/d |  | ≥20 g/d (Min. 132 g/w)^c^ |  | 3.9 (0.7-7.6) |  | 66 (6.9) |  |
| Milk and dairy products, g/d |  | ≥250 g/d |  | 197 (90-318) |  | 406 (37.1) |  |
| Meat and meat products, g/10 MJ |  | ≤50 g/10 MJ (Max. 350 g/w) |  | 110 (70-163) |  | 164 (15.0) |  |
|  |  |  |  |  |  |  |  |
| SFA, E% |  | <10 E% |  | 11.1 ± 2.7 |  | 382 (34.9) |  |
| Dietary fiber, g/MJ |  | ≥2 g/MJ |  | 2.7±0.7 |  | 900 (82.3) |  |
| Added sugar, E% |  | <10 E% |  | 8.2 (5.2-11.5) |  | 717 (65.5) |  |
| Iron, mg/d |  | ≥8 mg/d |  | 6.9 ± 2.2 |  | 302 (27.6) |  |
| Vit D supplement during winter^b^ |  | Supplement during winter (Oct-Apr) |  | 595 yes, 372 no |  | 595 (61.5) |  |
| Daily intakes are presented as median (IQR), mean±SD, and n when data were skewed, normally distributed, and binomial respectively.  ^a^Data was derived from the FFQ where n=780.  ^b^Data was derived from supplements’ questionnaire where n=794  ^c^The recommendation was recalculated to match the average energy intake of the included children. | | | | | | | |

| **Supplementary table 3.** Associations between sociodemographic characteristics and dietary intake among all participants with complete information on parental education and ethnic origin (n=1001) | | | | | | | | | | | | | | | | | | | | | | | | | | | | | | | | |  |
| --- | --- | --- | --- | --- | --- | --- | --- | --- | --- | --- | --- | --- | --- | --- | --- | --- | --- | --- | --- | --- | --- | --- | --- | --- | --- | --- | --- | --- | --- | --- | --- | --- | --- |
|  | Fruit and vegetables, g/ 10 MJ | | |  | Wholegrains, g/10 MJ | | |  | Fish^a^, g/d | | |  | Milk and dairy, g/10 MJ | | |  | Meat and meat products, g/10 MJ | | |  | Added sugar, E% | | |  | SFA, E% | | |  | Adherence score | | |  | |
|  | β  (95% CI) | | P |  | β  (95% CI) | | P |  | β  (95% CI) | | P |  | β  (95% CI) | | P |  | β  (95% CI) | | P |  | β  (95% CI) | | P |  | β  (95% CI) | | P |  | β  (95% CI) | | P |  | |
| **Sex** |  |  |  |  |  |  |  |  |  |  |  |  |  |  |  |  |  |  |  |  |  |  |  |  |  |  |  |  |  |  |  |  | |
| Girls | Ref. | | <0.001 |  | Ref. | | <0.001 |  | Ref. | | 0.987 |  | Ref. | | 0.724 |  | Ref. | | 0.031 |  | Ref. | | 0.021 |  | Ref. | | 0.579 |  | Ref. | | 0.416 |  | |
| Boys | -57.6  (-84.0, -31.2) | |  |  | 10.4  (4.9, 16.0) | |  |  | 0.0  (-2.0, 2.0) | |  |  | -5.4  (-27.1, 37.8) | |  |  | 9.8  (0.9, 18.7) | |  |  | -0.7  (-1.3, -0.1) | |  |  | -0.1  (-0.4, 0.2) | |  |  | 0.0  (-0.1, 0.2) | |  |  | |
|  |  | |  |  |  | |  |  |  | |  |  |  | |  |  |  | |  |  |  | |  |  |  | |  |  |  | |  |  | |
| **Age, y** | -29.0  (-51.3, -6.7) | | 0.011 |  | -2.9  (-7.6, 1.8) | | 0.225 |  | -1.8  (-3.5, -0.2) | | 0.032 |  | -59.2  (-86.6, 31.7) | | <0.001 |  | 4.3  (-3.2, 11.8) | | 0.264 |  | 0.2  (-0.3, 0.7) | | 0.411 |  | -0.1  (-0.4, 0.2) | | 0.539 |  | -0.2  (-0.3, -0.1) | | <0.001 |  | |
| **Weight status** |  |  |  |  |  | |  |  |  | |  |  |  | |  |  |  | |  |  |  | |  |  |  | |  |  |  |  |  |  | |
| Underweight | 5.6  (-49.7, 60.9) | | 0.894 |  | 7.8  (-3.9, 19.5) | | 0.284 |  | 2.6  (-1.7, 6.9) | | 0.284 |  | 18.9  (-49.2, 86.9) | | 0.810 |  | -18.6  (-37.2, 0.1) | | 0.026 |  | -0.4  (-1.6, 0.9) | | 0.065 |  | 0.3  (-0.4, 1.0) | | 0.060 |  | 0.0  (-0.2, 0.3) | | 0.805 |  | |
| Normal weight | Ref. | |  |  | Ref. | |  |  | Ref. | |  |  | Ref. | |  |  | Ref. | |  |  | Ref. | |  |  | Ref. | |  |  | Ref. | |  |  | |
| Overweight | -7.9  (-56.0, 40.2) | |  |  | -0.8  (-11.0, 9.4) | |  |  | 1.3  (-2.3, 5.0) | |  |  | -0.1  (-59.3, 59.1) | |  |  | 7.8  (-8.4, 24.0) | |  |  | -1.1  (-2.2, 0.0) | |  |  | -0.5  (-1.1, 0.1) | |  |  | 0.0  (-0.3, 0.2) | |  |  | |
| **Parental education** | |  |  |  |  |  |  |  |  |  |  |  |  |  |  |  |  |  |  |  |  |  |  |  |  |  |  |  |  |  |  |  | |
| Long | Ref. | | 0.144 |  | Ref. | | 0.007 |  | Ref. | | 0.036 |  | Ref. | | 0.743 |  | Ref. | | 0.064 |  | Ref. | | 0.314 |  | Ref. | | 0.826 |  | Ref. | | <0.001 |  | |
| Medium | -26.0  (-61.4, 9.4) | |  |  | -6.4  (-13.9, 1.1) | |  |  | -2.6  (-5.3, 0.1) | |  |  | 3.8  (-39.7, 47.4) | |  |  | 9.8  (-2.1, 21.8) | |  |  | 0.5  (-0.3, 1.3) | |  |  | -0.1  (-0.5, 0.4) | |  |  | -0.2  (-0.4, -0.1) | |  |  | |
| Short | -33.8  (-81.9, 14.3) | |  |  | -13.5  (-23.7, -3.3) | |  |  | -3.3  (-7.0, 0.4) | |  |  | 19.1  (-40.0, 78.3) | |  |  | 14.3  (-1.9, 30.5) | |  |  | 0.5  (-0.6, 1.6) | |  |  | 0.1  (-0.5, 0.7) | |  |  | -0.3  (-0.5, -0.1) | |  |  | |
| **Origin** |  |  |  |  |  |  |  |  |  |  |  |  |  |  |  |  |  |  |  |  |  |  |  |  |  |  |  |  |  |  |  |  | |
| Danish | Ref. | | 0.766 |  | Ref. | | <0.001 |  | Ref. | | 0.763 |  | Ref. | | 0.162 |  | Ref. | | 0.862 |  | Ref. | | <0.001 |  | Ref. | | 0.013 |  | Ref. | | 0.119 |  | |
| Non-Danish | 7.3  (-41.0, 55.6) | |  |  | -27.8  (-38.0, -17.6) | |  |  | 0.6  (-3.1, 4.3) | |  |  | 42.3  (-17.1, 101.8) | |  |  | -1.4  (-17.7, 14.8) | |  |  | -2.6  (-3.7, -1.5) | |  |  | 0.8  (0.2,1.4) | |  |  | -0.2  (-0.4, 0.0) | |  |  | |
| **Residential area** |  |  |  |  |  |  |  |  |  |  |  |  |  |  |  |  |  |  |  |  |  |  |  |  |  |  |  |  |  |  |  |  | |
| Urban | Ref. | | <0.001 |  | Ref. | | 0.020 |  | Ref. | | 0.349 |  | Ref. | | 0.333 |  | Ref. | | 0.162 |  | Ref. | | 0.001 |  | Ref. | | 0.014 |  | Ref. | | 0.019 |  | |
| Rural | -50.5  (-78.5, -22.5) | |  |  | 7.1  (1.1, 13.0) | |  |  | -1.0  (-3.1, 1.1) | |  |  | 17.0  (-17.5, 51.5) | |  |  | 6.7  (-2.7, 16.2) | |  |  | 1.1  (0.4, 1.7) | |  |  | 0.4  (0.1, 0.8) | |  |  | -0.2  (-0.3, 0.0) | |  |  | |
| Regression coefficients (β), 95% confidence intervals (CI), and *P*-values were derived from multivariable linear regression models examining the independent associations of sex, age, weight status, parental education level, country of origin, and residential area with intake of selected food groups and nutrients, as well as an overall dietary adherence score. ^a^Fish intake data were derived from the food frequency questionnaire, n=994 | | | | | | | | | | | | | | | | | | | | | | | | | | | | | | | | |  |

| **Supplementary table 4.** Associations between sociodemographic characteristics and dietary intake among all participants (n=1094) | | | | | | | | | | | | | | | | | | | | | | | | | | | | | | | | | |
| --- | --- | --- | --- | --- | --- | --- | --- | --- | --- | --- | --- | --- | --- | --- | --- | --- | --- | --- | --- | --- | --- | --- | --- | --- | --- | --- | --- | --- | --- | --- | --- | --- | --- |
|  | Fruit and vegetables, g/ 10 MJ | | |  | Wholegrains, g/10 MJ | | |  | Fish^a^, g/d | | |  | Milk and dairy, g/10 MJ | | |  | Meat and meat products, g/10 MJ | | |  | Added sugar, E% | | |  | SFA, E% | | |  | | Adherence score | | |  |
|  | β  (95% CI) | | P |  | β  (95% CI) | | P |  | β  (95% CI) | | P |  | β  (95% CI) | | P |  | β  (95% CI) | | P |  | β  (95% CI) | | P |  | β  (95% CI) | | P |  | β  (95% CI) | | | P |  |
| **Sex** |  |  |  |  |  |  |  |  |  |  |  |  |  |  |  |  |  |  |  |  |  |  |  |  |  |  |  |  |  | |  |  |  |
| Girls | Ref. | | <0.001 |  | Ref. | | <0.001 |  | Ref. | | 0.943 |  | Ref. | | 0.969 |  | Ref. | | 0.018 |  | Ref. | | 0.067 |  | Ref. | | 0.973 |  | Ref. | | | 0.446 |  |
| Boys | -55.1  (-80.4, -29.7) | |  |  | 11.6  (6.2, 17.1) | |  |  | -0.1  (-2.1, 1.9) | |  |  | 0.6  (-30.6, 31.9) | |  |  | 10.3  (1.7, 18.9) | |  |  | -0.5  (-1.1, 0.0) | |  |  | 0.0  (-0.3, 0.3) | |  |  | 0.0  (-0.1, 0.2) | | |  |  |
|  |  |  |  |  |  |  |  |  |  |  |  |  |  |  |  |  |  |  |  |  |  |  |  |  |  |  |  |  |  | |  |  |  |
| **Age, y** | -24.8  (-46.4, -3.3) | | 0.024 |  | -3.2  (-7.9, 1.4) | | 0.173 |  | -1.9  (-3.5, -0.2) | | 0.031 |  | -61.6  (-88.1, -35.0) | | <0.001 |  | 4.4  (-2.9, 11.7) | | 0.232 |  | 0.4  (-0.1, 0.9) | | 0.108 |  | -0.1  (-0.4, 0.1) | | 0.358 |  | -0.2  (-0.3, -0.1) | | | <0.001 |  |
| **Weight status** |  |  |  |  |  |  |  |  |  |  |  |  |  |  |  |  |  |  |  |  |  |  |  |  |  |  |  |  |  | |  |  |  |
| Underweight | 2.3  (-51.4, 56.0) | | 0.836 |  | 8.5  (-3.1, 20.1) | | 0.229 |  | 2.6  (-1.6, 6.9) | | 0.282 |  | 9.1  (-57.2, 75.3) | | 0.846 |  | -19.2  (-37.4, -1.0) | | 0.008 |  | -0.2  (-1.5, 1.0) | | 0.058 |  | 0.3  (-0.3,1.0) | | 0.094 |  | 0.1  (-0.2, 0.3) | | | 0.786 |  |
| Normal weight | Ref. | |  |  | Ref. | |  |  | Ref. | |  |  | Ref. | |  |  | Ref. | |  |  | Ref. | |  |  | Ref. | |  |  | Ref. | | |  |  |
| Overweight | -11.2  (-56.8, 34.3) | |  |  | 1.6  (-8.3, 11.4) | |  |  | 1.3  (-2.3, 4.9) | |  |  | -10.8  (-67.0, 45.4) | |  |  | 10.8  (-4.6, 26.2) | |  |  | -1.1  (-2.1, 0.0) | |  |  | -0.4  (-1.0, 0.2) | |  |  | 0.0  (-0.3, 0.2) | | |  |  |
| **Parental education** | |  |  |  |  |  |  |  |  |  |  |  |  |  |  |  |  |  |  |  |  |  |  |  |  |  |  |  |  | |  |  |  |
| Long | Ref. | | 0.268 |  | Ref. | | 0.009 |  | Ref. | | 0.058 |  | Ref. | | 0.476 |  | Ref. | | 0.166 |  | Ref. | | 0.567 |  | Ref. | | 0.303 |  | Ref. | | | 0.002 |  |
| Medium | -26.4  (-64.3, 11.4) | |  |  | -6.3  (-14.5, 1.8) | |  |  | -2.6  (-5.5, 0.2) | |  |  | 3.7  (-43.0, 50.4) | |  |  | 9.7  (-3.1, 22.5) | |  |  | 0.5  (-0.4, 1.4) | |  |  | -0.1  (-0.6, 0.4) | |  |  | -0.2  (-0.4, -0.1) | | |  |  |
| Short | -32.9  (-84.1, 18.4) | |  |  | -13.8  (-24.8, -2.7) | |  |  | -3.4  (-7.2, 0.5) | |  |  | 18.2  (-45.1, 81.4) | |  |  | 13.5  (-3.8, 30.9) | |  |  | 0.4  (-0.8, 1.6) | |  |  | 0.0  (-0.6, 0.7) | |  |  | -0.3  (-0.5, -0.1) | | |  |  |
| Unknown | -46.3  (-267.2, 174.5) | |  |  | 15.7  (-32.1, 63.5) | |  |  | -7.8  (-25.6, 9.9) | |  |  | 161.1  (-112.0, 434.2) | |  |  | 14.4  (-60.5, 89.4) | |  |  | 0.4  (-4.7, 5.5) | |  |  | 2.0  (-0.8, 4.7) | |  |  | -0.3  (-1.4, 0.8) | | |  |  |
| **Parental origin** |  |  |  |  |  |  |  |  |  |  |  |  |  |  |  |  |  |  |  |  |  |  |  |  |  |  |  |  |  | |  |  |  |
| Danish | Ref. | | 0.756 |  | Ref. | | <0.001 |  | Ref. | | 0.851 |  | Ref. | | 0.088 |  | Ref. | | 0.938 |  | Ref. | | <0.001 |  | Ref. | | 0.002 |  | Ref. | | | 0.407 |  |
| Non-Danish | 11.7  (-44.3, 67.8) | |  |  | -28.5  (-40.6, -16.4) | |  |  | 0.8  (-3.4, 5.0) | |  |  | 47.6  (-21.4, 116.6) | |  |  | -2.9  (-21.9, 16.0) | |  |  | -2.7  (-3.9, -1.4) | |  |  | 0.7  (0.0, 1.4) | |  |  | -0.2  (-0.4, 0.1) | | |  |  |
| Unknown | 57.3  (-148.5, 263.1) | |  |  | -20.8  (-65.3, 23.7) | |  |  | 5.7  (-29.6, 41.0) | |  |  | -145.1  (-398.8, 108.6) | |  |  | -2.9  (-72.6, 66.8) | |  |  | -1.2  (-6.0, 3.5) | |  |  | -2.7  (-5.2, -0.1) | |  |  | 0.0  (-2.2, 2.1) | | |  |  |
| **Residential area** | |  |  |  |  |  |  |  |  |  |  |  |  |  |  |  |  |  |  |  |  |  |  |  |  |  |  |  |  | |  |  |  |
| Urban | Ref. | | <0.001 |  | Ref. | | 0.027 |  | Ref. | | 0.375 |  | Ref. | | 0.313 |  | Ref. | | 0.121 |  | Ref. | | <0.001 |  | Ref. | | 0.003 |  | Ref. | | | 0.017 |  |
| Rural | -48.5  (-75.3, -21.7) | |  |  | 6.5  (0.7, 12.3) | |  |  | -1.0  (-3.1, 1.2) | |  |  | 17.0  (-16.1, 50.1) | |  |  | 7.2  (-1.9, 16.3) | |  |  | 1.2  (0.6, 1.8) | |  |  | 0.5  (0.2, 0.8) | |  |  | -0.2  (-0.3, 0.0) | | |  |  |
| Regression coefficients (β), 95% confidence intervals (CI), and *P*-values were derived from multivariable linear regression models examining the independent associations of sex, age, weight status, parental education level, country of origin, and residential area with intake of selected food groups and nutrients, as well as an overall dietary adherence score. ^a^Fish intake data were derived from the food frequency questionnaire, n=950 | | | | | | | | | | | | | | | | | | | | | | | | | | | | | | | | | |

| **Supplementary table 5.** Associations between sociodemographic characteristics and nutritional biomarkers among children with blood samples and complete data on parental education and ethnic origin | | | | | | | | | |
| --- | --- | --- | --- | --- | --- | --- | --- | --- | --- |
|  |  | Whole blood EPA+DHA^a^, FA% | | |  | Plasma total alkylresorcinols^b^ | | |  |
|  |  | β | 95% CI | P |  | β | 95% CI | P |  |
| **Sex** |  |  |  |  |  |  |  |  |  |
| Girls |  | Ref. | | 0.601 |  | Ref. | | 0.295 |  |
| Boys |  | -48 | -230, 133 |  |  | 1.09 | 0.93, 1.27 |  |  |
|  |  |  |  |  |  |  |  |  |  |
| **Age, y** |  | 147 | -3, 298 | 0.054 |  | 0.95 | 0.84, 1.08 | 0.462 |  |
|  |  |  |  |  |  |  |  |  |  |
| **Weight status** |  |  |  |  |  |  |  |  |  |
| Underweight |  | 17 | -379, 414 | 0.866 |  | 1.10 | 0.78, 1.55 | 0.663 |  |
| Normal weight |  | Ref. | |  |  | Ref. | |  |  |
| Overweight |  | 72 | -242, 385 |  |  | 1.09 | 0.83, 1.42 |  |  |
|  |  |  |  |  |  |  |  |  |  |
| **Parental education** |  |  |  |  |  |  |  |  |  |
| Long |  | Ref. | | 0.312 |  | Ref. | | 0.011 |  |
| Medium |  | -116 | -352, 119 |  |  | 0.82 | 0.67, 1.01 |  |  |
| Short |  | -204 | -565, 156 |  |  | 0.70 | 0.51, 0.97 |  |  |
|  |  |  |  |  |  |  |  |  |  |
| **Origin** |  |  |  |  |  |  |  |  |  |
| Danish |  | Ref. | | 0.344 |  | Ref. | | 0.745 |  |
| Non-Danish |  | 199 | -215, 613 |  |  | 0.94 | 0.66, 1.34 |  |  |
|  |  |  |  |  |  |  |  |  |  |
| **Residential area** |  |  |  |  |  |  |  |  |  |
| Urban |  | Ref. | | 0.002 |  | Ref. | | 0.273 |  |
| Rural |  | 337 | 123, 551 |  |  | 1.11 | 0.92, 1.34 |  |  |
| Regression coefficients (β), 95% confidence intervals (CI), and P-values were derived from multivariable linear regression models examining the independent associations of sex, age, weight status, parental education level, country of origin, and residential area with nutritional biomarkers of n-3 long-chain polyunsaturated fatty acids (fish) and wholegrain.  ^a^n=301  ^b^n=296. Plasma total alkylresorcinols were log-transformed for analysis and all estimates were back transformed to the original scale for presentation. | | | | | | | | | |

Children consented
(n=1350)

Children excluded from analyses due to:

No completed food diaries (n=208)

Parent registered own food intake instead of child’s (n=1)

Extreme values of energy intake (EI >15 MJ or EI <2 MJ) (n=10)

Missing height and/or weight measurement (n=38)

Children included in cross-sectional study
(n=1094)

Eligible children invited to participate
(n=2006)

24 schools agreed to participate

496 schools invited

1 school withdrew

**Supplementary fig. 1**: Participant flow chart


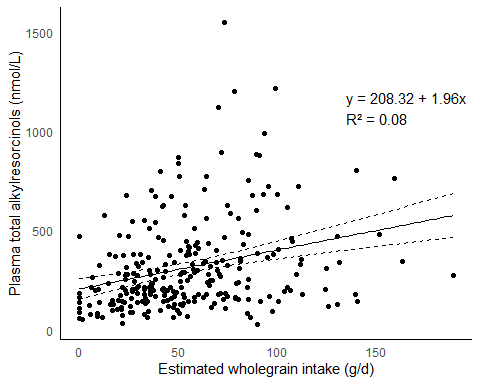


**Supplementary fig. 2.** Dose-response relationship between wholegrain intake and plasma total alkylresorcinols. The solid line represents the best-fitted regression line, and the dashed lines show the 95% confidence interval.


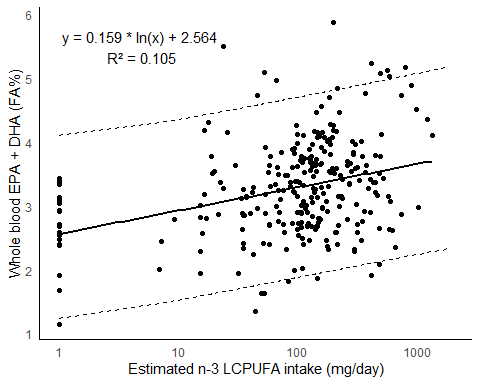


**Supplementary fig. 3.** Dose-response relationship between n-3 LCPUFA intake estimated by FFQ and whole blood EPA + DHA. The solid line represents the best-fitted regression line, and the dashed lines show the 95% confidence interval.
